# Supplementary material for: Risk factors for 3-month mortality in bedridden patients with hospital-acquired pneumonia: A multicentre prospective study
Source: PLoS One. 2021 Mar 30;16(3):e0249198. doi: 10.1371/journal.pone.0249198 (PMC8009424; doi:10.1371/journal.pone.0249198)
Supplement: Attachment — Submitted filename: Review of PONE-D-20-25311.docx [file pone.0249198.s001.docx]

Review of PONE-D-20-25311

In the manuscript “3-month mortality in bedridden patients with hospital-acquired pneumonia; a multicenter prospective study”, Jiao et al report the results from a secondary analysis of a prospective observational study that included 1141 patients diagnosed with HAP from 25 hospitals. They describe associations between patient characteristics, ward status, and treatments factors (surgical and anesthesia) versus inpatient mortality. They report ICU admission and patient comorbidities to be positively associated with death, and surgery with GA and tertiary hospital admission to be negatively associated with death, and conclude that the findings could help providers identify patients with high.

The etiology and consequences of HAP are poorly characterized, and a large prospective study of patients with a standard sampling method has much potential for adding knowledge. However, as currently written, the analysis seems to fall short of advancing our understanding dramatically, and the conclusion that using the risk factors for decision-making does not seem to be supported by the results. Some makor concerns:

Sampling method: While the case definition of a bedridden patient and HAP was well described, the process of enrollment into the prospective cohort was not immediately clear. Were patients identified and enrolled by treating clinicians? Was there a screening process applied to all hospitalized patients? If the latter, how was this process applied? The authors mention that patients with major complications of immobility at the time of enrollment were excluded; what were these exlclusion criteria? What was the enrollment % of patients initially screened?

Treatments during the hospitalization including ventilator use, antibiotic use, and glucocorticoid use were listed as patient charcteristics. I do not typically view these factors as patient characteristics, as they are interventions that might be associated with the other patient characteristics and the outcomes in very complex ways. If the study is intended to be a cohort study and these interventions are being examined as potential independent risk factors for HAP, then the timing is also incredibly important and I was unable to discern at what point in the hospitalization – before or after the development of HAP – these interventioned were identified. The results would have very different meaning to me depending on this.

Conclusions: It is hard to glean what meaning the authors feel this study has to our understanding from the conclusion. It is not surpirising that comorbidities are associated with higher mortality; however, whether the aCCI score has utility over clinical judgment is not supported in this study. It is als not surpiring that patients in the ICU have higher mortlity risk, but the authors’ causal explanations when attempting to explain some associations (ie, ICU admission leading to ventilator use leading to mortality, or surgical admission 🡪 differences in quality of medical care 🡪 lower mortality) seem very far-reaching. It all is further muddied by an unclear picture of how these patients were identified, and how that identification process accounted for – or did not – the potential sampling bias that renders the findings difficult to generalize.
