# Supplementary material for: Risk factors for 3-month mortality in bedridden patients with hospital-acquired pneumonia: A multicentre prospective study
Source: PLoS One. 2021 Mar 30;16(3):e0249198. doi: 10.1371/journal.pone.0249198 (PMC8009424; doi:10.1371/journal.pone.0249198)
Supplement: Attachment — Submitted filename: Response_letter_1223.docx [file pone.0249198.s002.docx]

**Response letter**

Dear Shane Patman,

Thank you for your letter and for the reviewers’ comments concerning our manuscript entitled “Risk factors for 3-month mortality in bedridden patients with hospital-acquired pneumonia: a multicentre prospective study” (ID: PONE-D-20-25311). Those comments were valuable and helpful for revising and improving the manuscript. We have taken the comments into careful consideration and have made corrections that we hope will be met with approval. Revised portions are marked in red in the paper. The main correction and the response to the reviewers’ comments are set out as below. We look forward to hearing from you regarding our submission. We would be glad to respond to any further questions and comments that you may have.

Journal Requirements:

When submitting your revision, we need you to address these additional requirements.

**1.) Please ensure that your manuscript meets PLOS ONE's style requirements, including those for file naming. The PLOS ONE style templates can be found at**

**https://journals.plos.org/plosone/s/file?id=wjVg/PLOSOne_formatting_sample_main_body.pdf and**

**https://journals.plos.org/plosone/s/file?id=ba62/PLOSOne_formatting_sample_title_authors_affiliations.pdf**

**Author response:** Thank you for the reminder. We have revised the documents according to the journal’s style requirements.

**2.) We suggest you thoroughly copyedit your manuscript for language usage, spelling, and grammar. If you do not know anyone who can help you do this, you may wish to consider employing a professional scientific editing service.  

Whilst you may use any professional scientific editing service of your choice, PLOS has partnered with both American Journal Experts (AJE) and Editage to provide discounted services to PLOS authors. Both organizations have experience helping authors meet PLOS guidelines and can provide language editing, translation, manuscript formatting, and figure formatting to ensure your manuscript meets our submission guidelines. To take advantage of our partnership with AJE, visit the AJE website (**[**http://learn.aje.com/plos/**](about:blank)**) for a 15% discount off AJE services. To take advantage of our partnership with Editage, visit the Editage website (www.editage.com) and enter referral code PLOSEDIT for a 15% discount off Editage services.  If the PLOS editorial team finds any language issues in text that either AJE or Editage has edited, the service provider will re-edit the text for free.**

**Upon resubmission, please provide the following:**

- **The name of the colleague or the details of the professional service that edited your manuscript**
- **A copy of your manuscript showing your changes by either highlighting them or using track changes (uploaded as a *supporting information* file)**
- **A clean copy of the edited manuscript (uploaded as the new *manuscript* file)**

**Author response:** Thank you for the reminder. The revised manuscript has been edited by AJE editors.

**3.) Thank you for stating in your financial disclosure:**

**'The funders had no role in study design, data collection and analysis, decision to publish, or preparation of the manuscript.'**

**At this time, please address the following queries:**

a. **Please clarify the sources of funding (financial or material support) for your study. List the grants or organizations that supported your study, including funding received from your institution.**
b. **State what role the funders took in the study. If the funders had no role in your study, please state: “The funders had no role in study design, data collection and analysis, decision to publish, or preparation of the manuscript.”**
c. **If any authors received a salary from any of your funders, please state which authors and which funders.**
d. **If you did not receive any funding for this study, please state: “The authors received no specific funding for this work.”**

**Please include your amended statements within your cover letter; we will change the online submission form on your behalf.**

**Author response:** Thank you for the suggestion. We have included amended statements in the cover letter.

**4.) We note that you have indicated that data from this study are available upon request. PLOS only allows data to be available upon request if there are legal or ethical restrictions on sharing data publicly. For information on unacceptable data access restrictions, please see http://journals.plos.org/plosone/s/data-availability#loc-unacceptable-data-access-restrictions.**

**In your revised cover letter, please address the following prompts:**

**a) If there are ethical or legal restrictions on sharing a de-identified data set, please explain them in detail (e.g., data contain potentially identifying or sensitive patient information) and who has imposed them (e.g., an ethics committee). Please also provide contact information for a data access committee, ethics committee, or other institutional body to which data requests may be sent.**

**b) If there are no restrictions, please upload the minimal anonymized data set necessary to replicate your study findings as either Supporting Information files or to a stable, public repository and provide us with the relevant URLs, DOIs, or accession numbers. Please see http://www.bmj.com/content/340/bmj.c181.long for guidelines on how to de-identify and prepare clinical data for publication. For a list of acceptable repositories, please see http://journals.plos.org/plosone/s/data-availability#loc-recommended-repositories.**

**We will update your Data Availability statement on your behalf to reflect the information you provide.**

**Author response:** Thank you for the reminder. We have added the statement in the updated cover letter.

**5.) Your ethics statement should only appear in the Methods section of your manuscript. If your ethics statement is written in any section besides the Methods, please delete it from any other section.**

**Author response:** Thank you for the reminder. We have confirmed that the ethics statement only appears in the Methods section.

**6.) We noticed you have some occurrence of overlapping text with the following previous publication(s), which needs to be addressed:**

**https://journals.plos.org/plosone/article?id=10.1371%2Fjournal.pone.0205729**

**In your revision ensure you cite all your sources (including your own works), and quote or rephrase any duplicated text outside the methods section. Further consideration is dependent on these concerns being addressed.**

**Author response:** Thank you for your important comments. We have carefully cited the references and revised the text thoroughly in the current study.

**Reviewers' comments:

Reviewer's Responses to Questions**

**Comments to the Author

1. Is the manuscript technically sound, and do the data support the conclusions?

The manuscript must describe a technically sound piece of scientific research with data that supports the conclusions. Experiments must have been conducted rigorously, with appropriate controls, replication, and sample sizes. The conclusions must be drawn appropriately based on the data presented.**

**Reviewer #1: Yes**

**Reviewer #2: Partly**

**2. Has the statistical analysis been performed appropriately and rigorously?**

**Reviewer #1: Yes**

**Reviewer #2: I Don't Know**

**3. Have the authors made all data underlying the findings in their manuscript fully available?

The** [**PLOS Data policy**](http://www.plosone.org/static/policies.action#sharing) **requires authors to make all data underlying the findings described in their manuscript fully available without restriction, with rare exception (please refer to the Data Availability Statement in the manuscript PDF file). The data should be provided as part of the manuscript or its supporting information, or deposited to a public repository. For example, in addition to summary statistics, the data points behind means, medians and variance measures should be available. If there are restrictions on publicly sharing data—e.g. participant privacy or use of data from a third party—those must be specified.**

**Reviewer #1: Yes**

**Reviewer #2: Yes**

**4. Is the manuscript presented in an intelligible fashion and written in standard English?

PLOS ONE does not copyedit accepted manuscripts, so the language in submitted articles must be clear, correct, and unambiguous. Any typographical or grammatical errors should be corrected at revision, so please note any specific errors here.**

**Reviewer #1: Yes**

**Reviewer #2: Yes**

**5. Review Comments to the Author

Please use the space provided to explain your answers to the questions above. You may also include additional comments for the author, including concerns about dual publication, research ethics, or publication ethics. (Please upload your review as an attachment if it exceeds 20,000 characters)**

**Reviewer #1: The authors identified the risk factors for mortality in bedridden patients with HAP during a 3-month observation period. They found that intensive care unit (ICU) admission, number of comorbidities and adjusted Charlson Comorbidity Index score were associated with an increased risk of mortality. However, several previous studies have shown similar results that disease severity and presence of comorbidities are associated with poor prognosis. The authors should clarify new evidences of this study.**

**Author response:** Thank you for your constructive and insightful advice about the findings. We agree that more evidence is needed to further clarify the study. We have included more patient-specific risk factors (including mobility at admission, immobility complications) and re-analysed the data. In the updated results, we additionally found that pressure injuries and VAP were risk factors for mortality among the population. The findings have great clinical implication for improving the prognosis of patients as they are modifiable factors. The detailed explanation and analysis of the results are described in the Discussion and Conclusion sections.

**Reviewer #2: In the manuscript “3-month mortality in bedridden patients with hospital-acquired pneumonia; a multicenter prospective study”, Jiao et al report the results from a secondary analysis of a prospective observational study that included 1141 patients diagnosed with HAP from 25 hospitals. They describe associations between patient characteristics, ward status, and treatments factors (surgical and anesthesia) versus inpatient mortality. They report ICU admission and patient comorbidities to be positively associated with death, and surgery with GA and tertiary hospital admission to be negatively associated with death, and conclude that the findings could help providers identify patients with high.

The etiology and consequences of HAP are poorly characterized, and a large prospective study of patients with a standard sampling method has much potential for adding knowledge. However, as currently written, the analysis seems to fall short of advancing our understanding dramatically, and the conclusion that using the risk factors for decision-making does not seem to be supported by the results. Some makor concerns:**

**Sampling method: While the case definition of a bedridden patient and HAP was well described, the process of enrollment into the prospective cohort was not immediately clear. Were patients identified and enrolled by treating clinicians? Was there a screening process applied to all hospitalized patients? If the latter, how was this process applied? The authors mention that patients with major complications of immobility at the time of enrollment were excluded; what were these exlclusion criteria? What was the enrollment % of patients initially screened?
Author response:** Thank you very much for your constructive advice about the sampling method. We apologize that this part was not described clearly in the original manuscript. We have revised the contents of this part of the manuscript (Page 3, line 140-151). The specific response to the questions you mentioned are listed as follows.

(1) The patients were identified and enrolled by pre-trained nurses. The screening process was only applied to bedridden patients (Page 3, line 140-144).

(2) The exclusion criteria were patients with more than one type of major complication of immobility (pressures injuries, deep vein thrombosis, and urinary tract infection) at the time of enrollment (Page 3, line 115-117).

(3) Regarding the enrollment percentage, we agree that it is an important indicator to the representativeness of the sample and have attempted to acquire the percentage. However, we failed because this was a secondary data analysis. We have mentioned this in the limitation section (Page 12, line 265-271).

**Treatments during the hospitalization including ventilator use, antibiotic use, and glucocorticoid use were listed as patient charcteristics. I do not typically view these factors as patient characteristics, as they are interventions that might be associated with the other patient characteristics and the outcomes in very complex ways. If the study is intended to be a cohort study and these interventions are being examined as potential independent risk factors for HAP, then the timing is also incredibly important and I was unable to discern at what point in the hospitalization – before or after the development of HAP – these interventioned were identified. The results would have very different meaning to me depending on this.
Author response:** Thank you very much for your careful review and constructive suggestions with regard to the risk factors. I am also grateful for the specific modification strategy. We agree that the treatment during hospitalization should not be viewed as patient characteristics, as they were affected by patients’ characteristics and may have impacted the outcomes in complicated ways. I have made comprehensive and detailed changes according to your suggestion. On one hand, in the risk factor candidates, we excluded the factors related to treatment, including ICU admission, ventilator use, antibiotic use, and glucocorticoid use. On the other hand, we further took more patient-specific factors into consideration, including mobility at admission and immobility complications (pressure injuries). We reanalysed the data and updated the results and discussions.

**Conclusions: It is hard to glean what meaning the authors feel this study has to our understanding from the conclusion. It is not surpirising that comorbidities are associated with higher mortality; however, whether the aCCI score has utility over clinical judgment is not supported in this study. It is als not surpiring that patients in the ICU have higher mortlity risk, but the authors’ causal explanations when attempting to explain some associations (ie, ICU admission leading to ventilator use leading to mortality, or surgical admission ◊ differences in quality of medical care ◊ lower mortality) seem very far-reaching. It all is further muddied by an unclear picture of how these patients were identified, and how that identification process accounted for – or did not – the potential sampling bias that renders the findings difficult to generalize.**

**Author response:** Thank you for your thoughtful comments. We agree that it is essential to make more specific and reasonable analyses and explanations on the relationship between the risk factors identified and mortality.

(1) In regard to comorbidities, previous studies on the comorbidities and mortality among pneumonia patients have mainly focused on PSI and CURB-65, both of which primarily use acute clinical findings to predict mortality. However, the role of chronic comorbidities in predicting outcomes is unclear. Therefore, we explored the ability of the ACCI to predict mortality in patients with HAP. We admit that more studies are needed on the performance and feasibility of ACCI. We have revised the discussion (Page 11, line 239-247).

(2) Regarding ICU admission, we excluded the variable in the multivariate analysis. As the reviewer suggested, it could not be a very good indicator to explain the mortality of HAP patients. In addition, ICU admission is also impacted by the patients’ decision making and total number of hospital ICU beds. In the updated results, we found that VAP increased the risk of mortality compared to non VAP patients. We have revised the discussion (Page 11, line 225-230).

**6. PLOS authors have the option to publish the peer review history of their article (**[**what does this mean?**](https://journals.plos.org/plosone/s/editorial-and-peer-review-process#loc-peer-review-history)**). If published, this will include your full peer review and any attached files.


If you choose “no”, your identity will remain anonymous but your review may still be made public.


Do you want your identity to be public for this peer review? For information about this choice, including consent withdrawal, please see our** [**Privacy Policy**](https://www.plos.org/privacy-policy)**.**

**Reviewer #1: Yes: Mutsuo Yamaya**

**Reviewer #2: No**

**[NOTE: If reviewer comments were submitted as an attachment file, they will be attached to this email and accessible via the submission site. Please log into your account, locate the manuscript record, and check for the action link "View Attachments". If this link does not appear, there are no attachment files.]

While revising your submission, please upload your figure files to the Preflight Analysis and Conversion Engine (PACE) digital diagnostic tool,** [**https://pacev2.apexcovantage.com/**](https://pacev2.apexcovantage.com/)**. PACE helps ensure that figures meet PLOS requirements. To use PACE, you must first register as a user. Registration is free. Then, login and navigate to the UPLOAD tab, where you will find detailed instructions on how to use the tool. If you encounter any issues or have any questions when using PACE, please email PLOS at** [**figures@plos.org**](mailto:figures@plos.org)**. Please note that Supporting Information files do not need this step.**
