# Supplementary material for: Risk factors for 3-month mortality in bedridden patients with hospital-acquired pneumonia: A multicentre prospective study
Source: PLoS One. 2021 Mar 30;16(3):e0249198. doi: 10.1371/journal.pone.0249198 (PMC8009424; doi:10.1371/journal.pone.0249198)
Supplement: Attachment — Submitted filename: Response_letter_0311.docx [file pone.0249198.s003.docx]

**Response letter**

Dear Shane Patman,

Thank you for your letter and for the reviewers’ comments concerning our manuscript entitled “Risk factors for 3-month mortality in bedridden patients with hospital-acquired pneumonia: a multicentre prospective study” (ID: PONE-D-20-25311). The comments we received were valuable and helped us in revising and improving our paper, as well as providing important guidance for our research. We carefully studied each comment and have made appropriate corrections, which we hope will meet with your approval. The revised text is marked in red in the manuscript. The main corrections in the text and our response to the editor’s comments are detailed below.

Once again, thank you for your comments and suggestions.

**Journal Requirements:**

**Please review your reference list to ensure that it is complete and correct. If you have cited papers that have been retracted, please include the rationale for doing so in the manuscript text, or remove these references and replace them with relevant current references. Any changes to the reference list should be mentioned in the rebuttal letter that accompanies your revised manuscript. If you need to cite a retracted article, indicate the article’s retracted status in the References list and also include a citation and full reference for the retraction notice.**
Author response: Thank you for the reminder. We have reviewed the reference list and ensured that it is completed and correct.

**Additional Editor Comments (if provided):**

**For this revision it was fortuitous to continue with the same peer reviewers from the initial submission. The sentiment from this review cycle is predominantly positive. Reviewer 2 notes an opportunity for slight adjustment to the manuscript discussion to enhance clarity and provide direction for future research efforts, as detailed below.**
Response：Thanks you for your positive comments. We have revised the discussion section according to the Reviewer’s suggestion.

[Note: HTML markup is below. Please do not edit.]

Reviewers' comments:

Reviewer's Responses to Questions

**Comments to the Author**

1. If the authors have adequately addressed your comments raised in a previous round of review and you feel that this manuscript is now acceptable for publication, you may indicate that here to bypass the “Comments to the Author” section, enter your conflict of interest statement in the “Confidential to Editor” section, and submit your "Accept" recommendation.

Reviewer #1: All comments have been addressed

Reviewer #2: All comments have been addressed

2. Is the manuscript technically sound, and do the data support the conclusions?

The manuscript must describe a technically sound piece of scientific research with data that supports the conclusions. Experiments must have been conducted rigorously, with appropriate controls, replication, and sample sizes. The conclusions must be drawn appropriately based on the data presented.

Reviewer #1: Yes

Reviewer #2: Yes

3. Has the statistical analysis been performed appropriately and rigorously?

Reviewer #1: Yes

Reviewer #2: Yes

4. Have the authors made all data underlying the findings in their manuscript fully available?

The [PLOS Data policy](http://www.plosone.org/static/policies.action#sharing) requires authors to make all data underlying the findings described in their manuscript fully available without restriction, with rare exception (please refer to the Data Availability Statement in the manuscript PDF file). The data should be provided as part of the manuscript or its supporting information, or deposited to a public repository. For example, in addition to summary statistics, the data points behind means, medians and variance measures should be available. If there are restrictions on publicly sharing data—e.g. participant privacy or use of data from a third party—those must be specified.

Reviewer #1: Yes

Reviewer #2: Yes

5. Is the manuscript presented in an intelligible fashion and written in standard English?

PLOS ONE does not copyedit accepted manuscripts, so the language in submitted articles must be clear, correct, and unambiguous. Any typographical or grammatical errors should be corrected at revision, so please note any specific errors here.

Reviewer #1: Yes

Reviewer #2: (No Response)

**6. Review Comments to the Author

Please use the space provided to explain your answers to the questions above. You may also include additional comments for the author, including concerns about dual publication, research ethics, or publication ethics. (Please upload your review as an attachment if it exceeds 20,000 characters)**

**Reviewer #1: (No Response)**

**Reviewer #2: Excellent revision. All of my major questions were addressed. One minor comment - authors discuss previous severity assessment tools (PSI/CURB-65), but these were intended for use to predict 30-day mortality in patients with community-onset pneumonia - it should probably be highlighted that these tools were never trained or designed for the use in patients with HAP. The lack of severity assessment tools specifically for this group of patients could rather be highlighted.**

Response: Thanks for your comments. Based on your helpful suggestions, we have made the necessary corrections to our previous draft. Detail of the corrections in the discussion is provided below.

With regard to the severity of illness, previous studies have mainly focused on Pneumonia Severity Index (PSI) and CURB-65 score, which were mainly used to predict mortality in patients with CAP [30] and were seldom trained or designed in patients with HAP [31]. The lack of severity assessment tools specifically for this group of patients has limited the ability to early detection of poor prognosis among HAP patients. The current study added new evidence by exploring the role of chronic comorbidities in predicting HAP patients’ outcomes. We found that the ACCI score was an independent risk factor for mortality among HAP patients. The ACCI is widely used to predict mortality and has been validated in various clinical populations [21, 32]. The results support the prognostic value of the ACCI score and indicate that the ACCI score may be useful for risk stratification and decision making of individual HAP patients. Future studies are necessary to compare its performance with CURB-65 and PSI and determine if this tool may be universally applied to HAP patients.

7. PLOS authors have the option to publish the peer review history of their article ([what does this mean?](https://journals.plos.org/plosone/s/editorial-and-peer-review-process#loc-peer-review-history)). If published, this will include your full peer review and any attached files.


If you choose “no”, your identity will remain anonymous but your review may still be made public.


**Do you want your identity to be public for this peer review?** For information about this choice, including consent withdrawal, please see our [Privacy Policy](https://www.plos.org/privacy-policy).

Reviewer #1: No

Reviewer #2: **Yes:** Barbara Jones

[NOTE: If reviewer comments were submitted as an attachment file, they will be attached to this email and accessible via the submission site. Please log into your account, locate the manuscript record, and check for the action link "View Attachments". If this link does not appear, there are no attachment files.]

While revising your submission, please upload your figure files to the Preflight Analysis and Conversion Engine (PACE) digital diagnostic tool, <https://pacev2.apexcovantage.com/>. PACE helps ensure that figures meet PLOS requirements. To use PACE, you must first register as a user. Registration is free. Then, login and navigate to the UPLOAD tab, where you will find detailed instructions on how to use the tool. If you encounter any issues or have any questions when using PACE, please email PLOS at [figures@plos.org](mailto:figures@plos.org). Please note that Supporting Information files do not need this step.
